# A new branch of understanding for barley inflorescence development

**DOI:** 10.1093/jxb/eraa464

**Published:** 2020-12-30

**Authors:** Kara A Levin, Scott A Boden

**Affiliations:** 1 School of Agriculture, Food and Wine, Waite Research Institute, University of Adelaide, Glen Osmond, Australia; 2 The John Innes Centre, Department of Crop Genetics, Norwich Research Park, Norwich, UK

**Keywords:** barley, development, inflorescence, palea, spikelet, TCP transcription factor

## Abstract

This article comments on:

**Shang Y, Yuan L, Di Y, Jia Y, Zhang Z, Li S, Xing L, Qi Z, Wang X, Zhu J, Hua W, Wu X, Zhu M, Li G, Li C**. 2020. A CYC/TB1 type TCP transcription factor controls spikelet meristem identity in barley. Journal of Experimental Botany **71**, 7118–7131.

**Genetic diversity for inflorescence architecture has helped improve the yields of our modern cereals. In barley, enhanced fertility of lateral spikelets has underpinned the generation of higher-yielding six-rowed cultivars, relative to their two-rowed progenitors. The genetic basis of lateral spikelet fertility has been well-characterised; however, very little is known of genes controlling other aspects of barley inflorescence architecture. Now,** Shang *et al.* (2020) **report on the identification of *BRANCHED AND INDETERMINATE SPIKELET 1* (*BDI1*), which encodes a CYC/TB1 type TCP transcription factor that represses inflorescence branching. In partnership with reports in rice and maize, these findings provide insights into genes controlling the diverse inflorescence architectures of cereals.**

## Moving beyond row-type architecture

Much of our knowledge of genes that regulate the arrangement of flowers in barley comes from investigating the development of spikelets, which are reproductive branches that form grain-producing florets (Gauley and Boden, 2019). In barley, spikelets are arranged in either two or six rows, with row-type determined by the lateral spikelets at each node being either sterile (two-row) or fertile (six-row) (Gauley and Boden, 2019). Wild barley species form two-row inflorescences, and defective alleles of at least five genes have been harnessed during breeding to improve fertility of the lateral spikelets, to generate six-rowed inflorescences (Gauley and Boden, 2019). These genes include *VRS1*, *VRS2*, *VRS3*, *VRS4* and *VRS5*: *VRS1* encodes a homeobox transcription factor that is expressed exclusively in lateral spikelets to suppress their development (Komatsuda *et al.*, 2007). *VRS3* and *VRS4* influence lateral spikelet fertility by promoting *VRS1* expression (Bull *et al.*, 2017; Koppolu *et al.*, 2013; van Esse *et al.*, 2017). *VRS5*, also known as *Intermedium-C* (*Int-C*), encodes a TCP transcription factor that is orthologous to TEOSINTE BRANCHED1 (TB1) from maize – *VRS5* suppresses development and outgrowth of the lateral spikelets (Ramsay *et al.*, 2011). TCPs form a large family of plant-specific transcription factors recognised by a bHLH motif, which are key regulators of differential plant form (Cubas *et al.*, 1999). This study, together with similar analyses in maize, rice and sorghum, raises the prospect that other TCP transcription factors contribute to distinct aspects of inflorescence development beyond row-type architecture (Bai *et al.*, 2012; Poursarebani *et al.*, 2020; Shang *et al.*, 2020; Yuan *et al.*, 2009).

Shang *et al.* (2020) interrogated a barley mutagenesis population to identify a mutant that forms extended branches at the base of the inflorescence. These branches are unique from the outgrowth of lateral spikelets that occur in the six-rowed cultivars, as they involve the central spikelet meristem maintaining an indeterminate state to produce a short secondary inflorescence or fused multi-florets. These branches resemble those of the barley *compositum2* (*com2*) mutant, where a mutation in an AP2/ERF transcription factor prevents termination of the spikelet meristem (Poursarebani *et al.*, 2015). Mutants such as *bdi1* and *com2*, therefore, provide an opportunity to investigate genetic pathways controlling the determinacy and fertility of the central spikelet within the triplet structure of barley, rather than lateral spikelets.

## Linking inflorescence development with palea formation

The major cereals, including barley, rice, maize, wheat and sorghum, display remarkable diversity in the arrangement of spikelets and florets that form on the inflorescence. Our understanding of the factors that contribute to inflorescence architecture diversity is improving as genes that control spikelet and floret development are discovered in different cereals, with certain genes shown to act as key regulators across multiple species. *BDI1*, for example, is an ortholog of *RETARDED PALEA1*/*DEFECTIVE BODY OF PALEA* (*REP1*/*DBOP*) in rice and *BRANCH ANGLE DEFECTIVE1* (*BAD1*) from maize, which were discovered by studying mutants that influence palea formation and development of lateral branches on the tassel, respectively (Bai *et al.*, 2012; Yuan *et al.*, 2009). The palea, together with the lemma, helps form a protective envelope for the floral organs, and in some species, contributes to determining maximum grain size (Lombardo and Yoshida, 2015). Curiously, *REP1* has no reported effect on inflorescence branching, nor does *BAD1* on palea development (Bai *et al.*, 2012; Yuan *et al.*, 2009). Taken together, the work on *BDI1* by Shang *et al.* (2020) and Poursarebani *et al.* (2020), who worked on the same gene that was named previously as *COMPOSITUM* (*COM1*), show that barley has potential to link the function of this TCP transcription factor in controlling palea formation and inflorescence branching across cereals (Poursarebani *et al.*, 2020) (Box 1).

Box 1. **Depiction of inflorescence architecture (brown) and developing floret phenotypes controlled by *BDI1/COM1* in barley and its orthologs in rice and maize**

**(A)** In wild-type barley, the developing inflorescence consists of one central determinate spikelet (CS), which contains a lemma (Le, blue) and a palea (Pa, red) that enclose floral organs. The *bdi1* mutant forms an indeterminate CS with extended branches, and thickened cell walls form within the palea (Poursarebani *et al.*, 2020). **(B)** In rice, a mutation in *OsREP1* forms a smaller palea (Yuan *et al.* 2009), but no known effects on panicle architecture have been shown. **(C)** In maize, a mutation in *ZmBAD1* causes defects in tassel branch angles, causing a clumped branching phenotype (Bai *et al.* 2012). No known effects of *bad1* alleles on palea development have been shown.

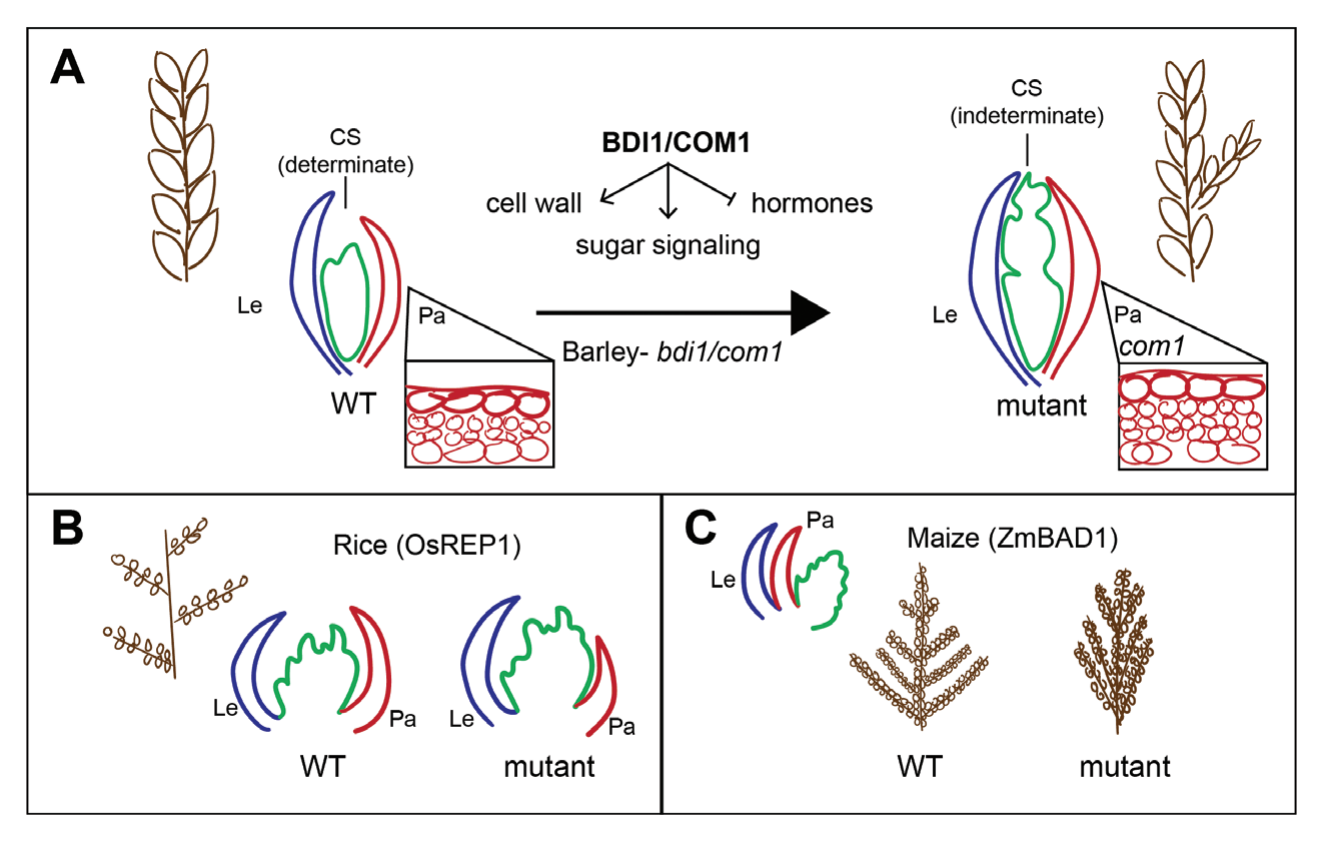

While the *bdi1* mutant is not reported to show palea-related phenotypes, except some fused florets sharing one palea (Shang *et al.*, 2020), certain *com1* mutants in a two-rowed cultivar (*cv.* Bowman) produced palea with increased cell size, cell wall thickness and more vascular bundles, relative to wild-type (Poursarebani *et al.*, 2020). These differences in palea-related phenotypes may be attributed to the different cultivars used in each study. Interestingly, the fused-grain phenotype of the *bdi1* mutant indicates that there may be a boundary organ defect during floret development, which might be consistent with the boundary signalling role described for *COM1* (Poursarebani *et al.*, 2020; Shang *et al.*, 2020). The effect of *com1* on palea cell size is the opposite of *rep1* in rice, in which cells are smaller; however, barley, rice, sorghum and Brachypodium mutants all produced more vascular bundles in the palea (Poursarebani *et al.*, 2020; Yuan *et al.*, 2009). No palea phenotype was reported in maize *bad1* mutants; however, the unique branched angle phenotype could be related to the positioning of the palea, which is located on the same side as the lemma (Box 1) (Bai *et al.*, 2012; Lombardo and Yoshida, 2015). Given the palea is proposed to act as a sink for auxin, and perturbed auxin distribution is associated with altered spikelet architecture, BDI1/COM1 may influence spikelet architecture and floret development by modifying auxin-related processes during early inflorescence development (Bartlett *et al.*, 2015; Boden, 2017; Yang *et al.*, 2017; Youssef *et al.*, 2017). BDI1/COM1 function should therefore be investigated further to understand the link between palea development and spikelet architecture in cereals.

## Regulation of spikelet determinacy by cell walls and trehalose metabolism

The transcriptome analysis performed by Shang *et al.* (2020) points towards molecular processes that contribute to the inflorescence branching of *bdi1*. During early inflorescence development, *bdi1* mutants showed altered expression of genes involved in cell wall development, hormone signalling and carbohydrate-based metabolic processes (Shang *et al.*, 2020). The effect on cell wall development genes is consistent with the phenotypes and transcriptome changes observed in *com1* mutants, and indicates that cell boundary formation plays a key role in regulating spikelet meristem determinacy (Poursarebani *et al.*, 2020). Of the genes involved in carbohydrate metabolism, a gene encoding a trehalose 6-phosphate phosphatase (known as *SISTER OF RAMOSA3*; *SRA3*) is down-regulated in *bdi* and *com1*, relative to wild-type (Koppolu *et al.*, 2013; Poursarebani *et al.*, 2020; Satoh-Nagasawa *et al.*, 2006; Shang *et al.*, 2020). The lower expression of *SRA3* and its association with inflorescence branching are consistent with the reduced spikelet meristem determinacy of *ramosa3* and *tpp4* mutants of maize (Claeys *et al.*, 2019; Satoh-Nagasawa *et al.*, 2006). Together, these results highlight the importance of further investigating cell wall development and trehalose metabolism to enhance our understanding of yield-related traits in barley.

In conclusion, the study by Shang *et al.* (2020) joins an emerging list of publications reporting the identification of genes controlling inflorescence development in barley, beyond those that control row-type architecture. These studies provide an ideal opportunity to compare gene function with that of the more-studied rice and maize, and to improve our understanding of the underlying genetic pathways contributing to the remarkable diversity of inflorescence architecture among cereals.
